# Predicting response to neoadjuvant chemotherapy with liquid biopsies and multiparametric MRI in patients with breast cancer

**DOI:** 10.1038/s41523-024-00611-z

**Published:** 2024-01-20

**Authors:** L. M. Janssen, M. H. A. Janse, B. B. L. Penning de Vries, B. H. M. van der Velden, E. J. M. Wolters-van der Ben, S. M. van den Bosch, A. Sartori, C. Jovelet, M. J. Agterof, D. Ten Bokkel Huinink, E. W. Bouman-Wammes, P. J. van Diest, E. van der Wall, S. G. Elias, K. G. A. Gilhuijs

**Affiliations:** 1 Image Sciences Institute, University Medical Centre Utrecht, Utrecht University, Utrecht, The Netherlands; 2 Julius Center for Health Sciences and Primary Care, University Medical Centre Utrecht, Utrecht University, Utrecht, The Netherlands; 3 Department of Radiology, St Antonius Hospital, Nieuwegein, The Netherlands; 4 Philips Research, Eindhoven, The Netherlands; 5 Agena Bioscience GmbH, Hamburg, Germany; 6 Stilla Technologies, Villejuif, France; 7 Department of Medical Oncology, St. Antonius Hospital, Nieuwegein, The Netherlands; 8 Department of Medical Oncology, Alexander Monro Hospital, Bilthoven, The Netherlands; 9 Department of Medical Oncology, Albert Schweitzer Hospital, Dordrecht, The Netherlands; 10 Department of Pathology, University Medical Centre Utrecht, Utrecht University, Utrecht, The Netherlands; 11 Department of Medical Oncology, University Medical Centre Utrecht, Utrecht University, Utrecht, The Netherlands

**Keywords:** Tumour biomarkers, Predictive markers

## Abstract

Accurate prediction of response to neoadjuvant chemotherapy (NAC) can help tailor treatment to individual patients’ needs. Little is known about the combination of liquid biopsies and computer extracted features from multiparametric magnetic resonance imaging (MRI) for the prediction of NAC response in breast cancer. Here, we report on a prospective study with the aim to explore the predictive potential of this combination in adjunct to standard clinical and pathological information before, during and after NAC. The study was performed in four Dutch hospitals. Patients without metastases treated with NAC underwent 3 T multiparametric MRI scans before, during and after NAC. Liquid biopsies were obtained before every chemotherapy cycle and before surgery. Prediction models were developed using penalized linear regression to forecast residual cancer burden after NAC and evaluated for pathologic complete response (pCR) using leave-one-out-cross-validation (LOOCV). Sixty-one patients were included. Twenty-three patients (38%) achieved pCR. Most prediction models yielded the highest estimated LOOCV area under the curve (AUC) at the post-treatment timepoint. A clinical-only model including tumor grade, nodal status and receptor subtype yielded an estimated LOOCV AUC for pCR of 0.76, which increased to 0.82 by incorporating post-treatment radiological MRI assessment (i.e., the “clinical-radiological” model). The estimated LOOCV AUC was 0.84 after incorporation of computer-extracted MRI features, and 0.85 when liquid biopsy information was added instead of the radiological MRI assessment. Adding liquid biopsy information to the clinical-radiological resulted in an estimated LOOCV AUC of 0.86. In conclusion, inclusion of liquid biopsy-derived markers in clinical-radiological prediction models may have potential to improve prediction of pCR after NAC in breast cancer.

## Introduction

Neoadjuvant chemotherapy (NAC) is often used in the treatment of patients with early-stage breast cancer. Benefits of the neoadjuvant strategy include the possibility for more conservative surgery of breast and axilla, while resulting in comparable rates of distant recurrence and overall survival (OS) when compared to adjuvant chemotherapy^1–3^. The neoadjuvant approach leaves the tumor in situ, allowing assessing the effectiveness of treatment. The degree of response to NAC is known to vary between the different breast cancer subtypes and treatments, with rates of pathologic complete response (pCR) ranging from 8% to 68%^4–6^. Achieving pCR is related to better overall outcome (hazard ratio for OS for pCR versus no pCR: 0.36 (95% confidence interval (CI) 0.31–0.42), as reported by ref. ^4^. However, the prognostic value of pCR can vary depending on the definition of pCR and the tumor subtype.

During NAC, the response of the tumor to treatment can be assessed by imaging methods such as magnetic resonance imaging (MRI). According to clinical guidelines, radiological assessment of MRI by Response Evaluation Criteria in Solid Tumors (RECIST)^7^ only leads to treatment changes if a clear progression is visible^8^. Because residual disease cannot be reliably ruled out by radiological assessment of MRI alone^9^, an unmet clinical need exists for non-invasive methods to guide treatment decisions. A promising approach for obtaining more information from MRI is to use computer-extracted features to predict treatment response in addition to traditional radiological assessment^10–13^.

Another approach to assess response to NAC that is currently the focus of intensive research is the use of liquid biopsies, in which body fluids such as blood are analyzed for the presence of cell-free DNA (cfDNA) that can be characterized as circulating tumor DNA (ctDNA) using various methods. The detection of ctDNA before, during and after NAC has previously been shown to be useful in predicting and monitoring response to NAC^14–16^. There has been little research on the combination of computer extracted MRI features and liquid biopsies for predicting response to NAC, especially in addition to traditional predictors of response, such as tumor grade, nodal status, receptor subtype and radiological response.

Chemotherapy can lead to serious adverse effects^17,18^. In the treatment of early-stage breast cancer, physicians must, therefore, find the balance between risks of overtreatment with its associated side effects and undertreatment with the potential for inadequate disease control. Improving the ability to predict a patients response to treatment is essential for tailoring the treatment to the needs of each patient and de-escalate treatment while maintaining oncological safety.

An opportunity that comes with neoadjuvant treatment is the adaptation of treatment based on the observed response of the tumor that remains in situ during therapy. This may involve switching to another treatment when the tumor regresses insufficiently, de-escalating or even discontinuing treatment when a complete response occurs. Accurate, non-invasive methods for predicting treatment response are essential in each of these scenarios.

The current explorative study aims to investigate the potential of combining computer-extracted MRI features and liquid biopsies derived before, during and after NAC with traditional markers for response to NAC in a prospective multicenter clinical study.

## Results

### Patient characteristics

The first patient was included on January 2nd, 2020, and the last visit of the last patient was on May 16th, 2022. A total of 61 patients enrolled instead of the intended 100 patients, which was due to the COVID-19 pandemic as well as the time constrains pertaining to the funding conditions.

The characteristics of these patients and their tumors are summarized in Table 1. Twenty-three of 61 (38%) patients achieved pCR after completion of NAC.

**Table 1:** Characteristics of 61 included early-stage breast cancer patients and their tumors, treated with NAC and surgery in 4 Dutch hospitals.

		Total (*N* = 61)
Age (years)	Median (Min, Max)	50.0 (25, 72)
Histology	Invasive carcinoma NST	53 (86.9%)
	Ductolobular carcinoma	2 (3.3%)
	Lobular carcinoma	5 (8.2%)
	Mucinous carcinoma	1 (1.6%)
Grade	2	24 (39.3%)
	3	37 (60.7%)
Receptor subtype	ER−/HER2−	21 (34.4%)
	ER + −/HER2+	17 (27.9%)
	ER + /HER2−	23 (37.7%)
cT stage	T1	11 (18.3%)
	T2	37 (61.7%)
	T3	12 (20.0%)
	Missing	1
Nodal metastases	Absent	23 (37.7%)
	Present	38 (62.3%)
Neoadjuvant treatment	Paclitaxel, trastuzumab, carboplatin and pertuzumab	16 (26.2%)
	Doxorubicin and paclitaxel	26 (42.6)
	Doxorubicin, paclitaxel and carboplatin	18 (29.5%)
	Paclitaxel and trastuzumab	1 (1.6%)
Radiologist conclusion MRI on-treatment	Partial response	47 (77.0%)
	Radiological complete remission	9 (14.8%)
	No response	4 (6.6%)
	Missing	1 (1.6%)
Radiologist conclusion MRI post-treatment NAC	Partial response	21 (34.4%)
	Radiological complete remission	36 (59.0%)
	No response	2 (3.3%)
	Missing	2 (3.3%)
Surgery type	Mastectomy	36 (59.0%)
	Lumpectomy	25 (41.0%)
Pathological complete response	Yes No	23 (38%) 38 (62%)
Residual Cancer Burden	Median (Min, Max)	1.254 (0, 3.42)

*ER* estrogen receptor, *HER2* Human epidermal growth factor receptor-2.

A total of 493 blood samples were collected (Supplementary Fig. 1). Ten mutations and five HER2 amplifications were found in 286 samples evaluable for mutation analysis (Supplementary Table 1).

### Prediction models based on clinical and clinical-radiological features

Table 2 lists the performance of each model, expressed by the area under the curve (AUC), including their 95% naïve CI, calculated by L1-penalized maximum likelihood estimation (LASSO) and Receiver Operator Characteristic (ROC) curve analysis and internally validated by leave-one-out cross validation (LOOCV). The mean squared error (MSE) can be found in Supplementary Table 2, coefficients of each model in Supplementary Table 3 and ROC curves and calibration plots in Supplementary Figs. 2 and 3.

**Table 2:** Summary of model performance corrected for optimism by LOOCV including naive confidence intervals.

	Pre-treatment	On-treatment	Post-treatment
	AUC (95% CI)	AUC (95% CI)	AUC (95% CI)
Clinical-radiological	0.76 (0.62–0.88)	0.83 (0.71–0.93)	0.82 (0.71–0.92)
Liquid biopsies	NA	0.51 (0.34–0.67)	0.76 (0.63–0.88)
MRI features	NA	0.66 (0.51–0.81)	0.69 (0.53–0.81)
Clinical-radiological + liquid biopsies	0.77 (0.64–0.88)	0.82 (0.71–0.92)	0.86 (0.76–0.94)
Clinical-radiological + MRI features	0.77 (0.64–0.88)	0.82 (0.70–0.92)	0.83 (0.70–0.93)
Liquid biopsies + MRI features	NA	0.64 (0.47–0.78)	0.76 (0.64–0.88)
Clinical-radiological + liquid biopsies + MRI features	0.76 (0.63–0.88)	0.81 (0.70–0.91)	0.86 (0.75–0.94)
Only clinical	0.76 (0.62–0.87)	0.76 (0.62–0.87)	0.76 (0.62–0.87)
Only clinical + liquid biopsies	0.76 (0.63–0.87)	0.78 (0.65–0.88)	0.85 (0.74–0.94)
Only clinical + MRI features	0.76 (0.63–0.87)	0.82 (0.71–0.91)	0.84 (0.72–0.93)
Only clinical + MRI features + liquid biopsies	0.76 (0.63–0.87)	0.81 (0.70–0.91)	0.85 (0.74–0.93)

*CI* confidence interval, *AUC* area under the receiver operating characteristic curve to discriminate between pCR (RCB 0) and non-pCR, *NA* not applicable (no predictors remained in the model).

In the pre-treatment clinical-radiological model, the maximum tumor diameter on MRI did not contribute enough and was therefore removed from the optimal model. As a result, the pre-treatment clinical and the clinical-radiological models were identical, yielding an estimated LOOCV AUC of 0.76 (95% CI 0.62–0.88).

The estimated LOOCV AUC of the on-treatment clinical-radiological model increased to an estimated LOOCV AUC of 0.83 (95% CI 0.71–0.93). The performance of the post-treatment model did not increase further, the estimated LOOCV AUC remaining at 0.82 (95% CI 0.71–0.92).

### Prediction models based on computer extracted MRI features

In the optimal pre-treatment prediction model, based only on MRI features, no variables were selected. In the on-treatment model, only the ensemble of volume features remained in the model, resulting in an estimated LOOCV AUC of 0.66 (95% CI 0.51–0.81). The predictors contributing to the optimal post-treatment model were ensembles of tumor volume features, T2 features and tumor diameter features, yielding an estimated LOOCV AUC of 0.69 (95%CI 0.53–0.81).

### Prediction models based on liquid biopsies

No liquid biopsy predictors were informative enough to contribute to a pre-treatment model. The on-treatment liquid biopsy model included the ensemble of the total amount of cfDNA, resulting in an estimated LOOCV AUC of 0.51 (95% CI 0.34–0.67). The post-treatment model incorporated ensembles of both the total amount of cfDNA and methylation, resulting in an estimated LOOCV AUC of 0.76 (95% CI 0.63–0.88). Specifically, increases in methylated *AKR1B1*, *HIST1H3C* and *TM6SF1* during treatment were found to be correlated with a higher Residual Cancer Burden (RCB) (Supplementary Fig. 4).

### Combined prediction models

Incorporating the post-treatment liquid biopsy model into the post-treatment clinical-radiological model led to a higher estimated LOOCV AUC compared to that from the post-treatment clinical-radiological model alone (estimated LOOCV AUC 0.86 (95% CI 0.76–0.94) vs. 0.82 (95% CI 0.71–0.92)).

The post-treatment only-clinical + liquid biopsies model yielded higher estimated LOOCV AUC compared to the post-treatment clinical-radiological model. The on-treatment only clinical + MRI features model also led to higher estimated LOOCV AUC than the on-treatment and post-treatment clinical-only models. Additionally, the on-treatment and post-treatment only clinical + MRI features model showed comparable estimated LOOCV AUC as the clinical-radiological models (Table 2).

The combination of pre-treatment and on-treatment clinical-radiological models with liquid biopsies and computer extracted MRI features did not indicate higher AUC’s compared to either model alone. Adding MRI features to the post-treatment clinical-radiological or liquid biopsy model did not lead to a higher estimated LOOCV AUC. Furthermore, at none of the time points did the combination of liquid biopsy and MRI features (without clinical-radiological predictors) lead to a higher estimated LOOCV AUC compared to either model alone.

## Discussion

Improving methods for the prediction of response to NAC is essential for personalizing treatment of early breast cancer and ultimately reducing unnecessary side-effects without negatively affecting a patient’s outcome. The potential for an improved prediction by combining liquid biopsies and computer extracted features of multiparametric MRI with known clinical predictors had not yet been established in the literature. Here, we aimed to explore this combination in relation to response to NAC in breast cancer in a prospective multicenter clinical study.

Our results suggest that incorporating liquid biopsies after NAC into a clinical-radiological prediction model is informative of pCR after NAC (estimated LOOCV AUC 0.86 (95% CI 0.76–0.94) with liquid biopsies versus 0.82 (95%CI 0.71–0.92) without liquid biopsies). Notably, post-treatment liquid biopsies alone were found to have some association with pCR, but not pre-treatment or on-treatment liquid biopsies.

**Fig. 1:**
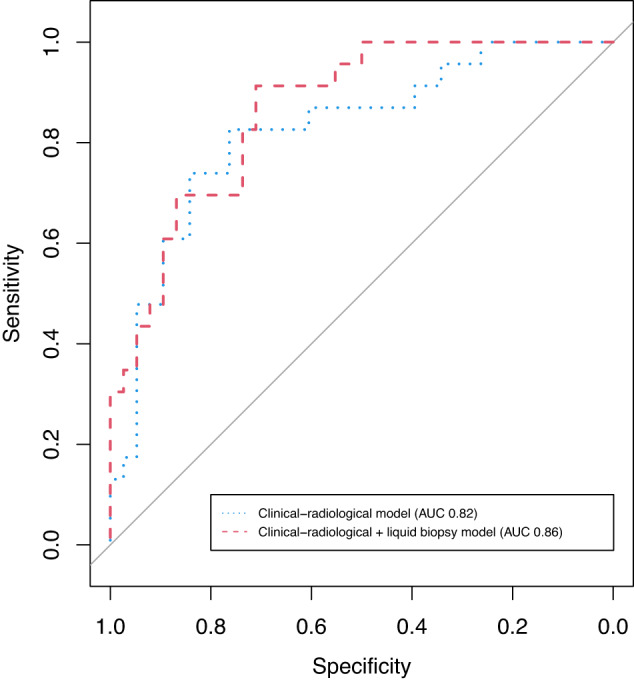
Receiver operating characteristic curves of combined prediction models at time point 3. Red dashed curve represents the clinical-radiological + liquid biopsy model, resulting in an estimated LOOCV AUC of 0.86. The blue dotted curve represents the clinical-radiological model, corresponding to an estimated LOOCV AUC of 0.82.

The shape of the ROC curve of the post-treatment clinical-radiological-liquid biopsy model (Fig. 1) suggests higher sensitivity at high specificity levels compared to the clinical-radiological model, although this should be interpreted cautiously given the limited sample size. A high specificity (defined as the proportion of patients with residual disease that is correctly classified as such) and positive predictive value (the proportion of patients that the model predicts will have pCR and who do indeed have pCR at pathologic assessment) is essential for selecting patients for safe de-escalation of (surgical) treatment in the future (i.e., a watch-and-wait approach, sparing patients surgery-associated morbidity or de-escalation of adjuvant systemic treatment). Nonetheless, if the sensitivity of the model (defined as the proportion of patients with pCR who are correctly classified as such) is very low, too few patients can be selected, which will make the watch-and-wait approach of limited value in clinical practice.

In our study, most predictive information from the liquid biopsies seemed to come from the total amount of cfDNA and the methylation markers, while mutation status of usual suspect breast cancer genes and integrity of the cfDNA did not hold much information. Specifically, in our study, an increase in methylation of *AKR1B1*, *HIST1H3C* or *TM6SF1* during treatment was found to be correlated with a higher RCB (Supplementary Fig. 4). This finding aligns with the findings of a previous study by Fackler et al. on metastatic breast cancer. In that study, the researchers found that a decrease in these markers was observed in patients who responded to treatment, but not in patients who did not respond^19^. Studies in early breast cancer have also shown the potential of other methylation markers in blood to predict the response to NAC^20–22^. Our results add to the existing evidence that methylation could be a promising biomarker to focus on in future research for response monitoring of neoadjuvant treatment using liquid biopsies.

Our results also show that the estimated discriminative ability of the post-treatment clinical + liquid biopsies model, as measured by the LOOCV AUC, is slightly higher compared to the post-treatment clinical-radiological model (Table 2). This suggests that liquid biopsies may be a reasonable alternative for evaluating response in patients who cannot undergo MRI due to contra-indications such as presence of metallic implants.

On-treatment and post-treatment computer-extracted MRI features were found to be associated with response. We also estimated higher LOOCV AUC when these computer-extracted MRI features were combined with clinical variables, compared to the estimated LOOCV AUC of a clinical-only model. The combined model had a similar estimated LOOCV AUC as the on-treatment and post-treatment clinical-radiological model (Table 2). Adding the computer-extracted features to the clinical-radiological model did not lead to obvious improvement of performance. An evident benefit of these features as an addition to, or replacement of, traditional radiological assessment was thus not observed in our study with a limited sample size.

The combination of liquid biopsy and computer extracted MRI features without clinical-radiological predictors did not show a higher estimated LOOCV AUC compared to either model alone at any of the time points. This suggests no indication for complementary value of these two technologies in our study. The study by Magbanua et al. suggested that functional tumor volume (FTV) on MRI and ctDNA are correlated measures of tumor burden^23^. For the time points before, halfway and after NAC, Magbanua et al. did not find any complementary value of ctDNA in addition to FTV for the prediction of pCR either. They did find a non-significant increase in AUC from 0.59 to 0.69 when adding ctDNA after 3 weeks to FTV (including IHC subtype, but no other clinical variables) for the prediction of pCR, whereas in this study no prediction was made after 3 weeks^16^.

Our study is limited by its sample size, which was smaller than expected due to enrollment issues, limiting the power. The sample size enabled only observations of large effects, thus limiting the detection of features with smaller effects which still can be clinically relevant. In follow-up studies, a larger sample size would be beneficial. We found very few mutations overall, which may be due to the use of a generic panel (instead of a personalized tissue-based panel). This could have led to an underestimation of the predictive value of ctDNA mutations. However, most somatic mutations (except p53 and PIK3CA) are infrequent in early stage breast cancer because of the significant molecular heterogeneity^24^, so even with a tissue based panel, the number of detected mutations could have been limited. We decided that a simpler generic panel would require less resources and allow easier translation into the clinical workflow. Another limitation in our study is the lack of external validation, which may hamper translation of our models to other patient cohorts. To mitigate this issue, we used rigorous cross-validation with an inner and outer loop and employed L1-penalized maximum likelihood estimation (LASSO) to obtain the most parsimonious model with the least number of parameters. External validation of our results in independent larger cohorts is, however, still required. Larger follow-up studies should further investigate the potential of methylation in liquid biopsies as a biomarker to rule out residual disease after NAC, thus ultimately designing trials on omitting surgery safely. Trials on omission of surgery based on pCR in tissue biopsy have been proposed, and the results of one small study seem promising^25,26^. However, the invasive nature of multiple tissue biopsies may make liquid biopsies the more patient-friendly option, which is why we opted for this approach. A disadvantage is, however, that liquid biopsy analysis is typically not yet implemented in daily clinical practice and may, therefore, not be readily available, as opposed to tissue biopsy. This could make the translation into daily practice more challenging.

Our findings could motivate future research on liquid biopsies as an alternative to MRI for response evaluation in patients with contra-indications for MRI. Future research should also focus on new methods to improve response prediction before and during neoadjuvant treatment, in order to eventually be able to guide de-escalation of systemic therapy.

In conclusion, our results suggest that adding liquid-biopsy derived amount of cfDNA and methylation markers to clinical-radiological prediction models is informative of pCR after NAC. Our results also suggest a positive contribution of liquid biopsies towards assessment of tumor response compared to radiological assessment of MRI in combination with a post-treatment clinical model. Furthermore, a model combining computer-extracted MRI features and clinical variables performed equally well compared to a model with radiological assessment of MRI combined with clinical variables during and after NAC. We were not able to detect increased association with response by combining computer extracted MRI features and liquid biopsies.

## Methods

### Study design

The LIMA study is a prospective multicenter observational study in patients with breast cancer undergoing NAC, following the protocol previously described^27^. In short, patients undergoing NAC were monitored using longitudinal multiparametric MRI and liquid biopsies (blood).

All patients signed informed consent before enrollment. The study was conducted in accordance with the Declaration of Helsinki and approved by the Medical Ethics Review Committee of the University Medical Center Utrecht (19–396, NL67308.041.19).

Inclusion criteria were: Female patients aged 18 years or older, histologically proven invasive breast carcinoma and planned to receive NAC. Exclusion criteria were: patients with estrogen receptor (ER)-positive and HER2-negative breast cancer tumors that were also Bloom and Richardson grade 1, patients with inflammatory breast cancer, distant metastases on positron emission tomography/computed tomography (PET/CT), prior ipsilateral breast cancer (contralateral breast cancer >5 years ago allowed), other active malignant diseases in the past 5 years (excluding squamous cell or basal cell carcinoma of the skin), pregnancy or lactation, contra-indications for MRI according to standard hospital guidelines, contra-indications for gadolinium-based contrast-agent, including known prior allergic reaction to any contrast-agent, and renal failure, defined by a glomerular filtration rate <30 mL/min/1.73 m^2^.

All patients underwent NAC according to Dutch guidelines^8^. Treatment consisted of 4 cycles adriamycin and cyclophosphamide followed by 12 times weekly paclitaxel (AC-P) with or without carboplatin for patients with HER2-negative tumors. For HER2-positive tumors treatment consisted of 9 cycles of pertuzumab, trastuzumab, carboplatin and paclitaxel (PTCP), or, if low-risk disease, the Tolaney schedule consisting of 12 cycles of weekly paclitaxel and trastuzumab^28^.

### Study procedures and endpoint

**Fig. 2:**
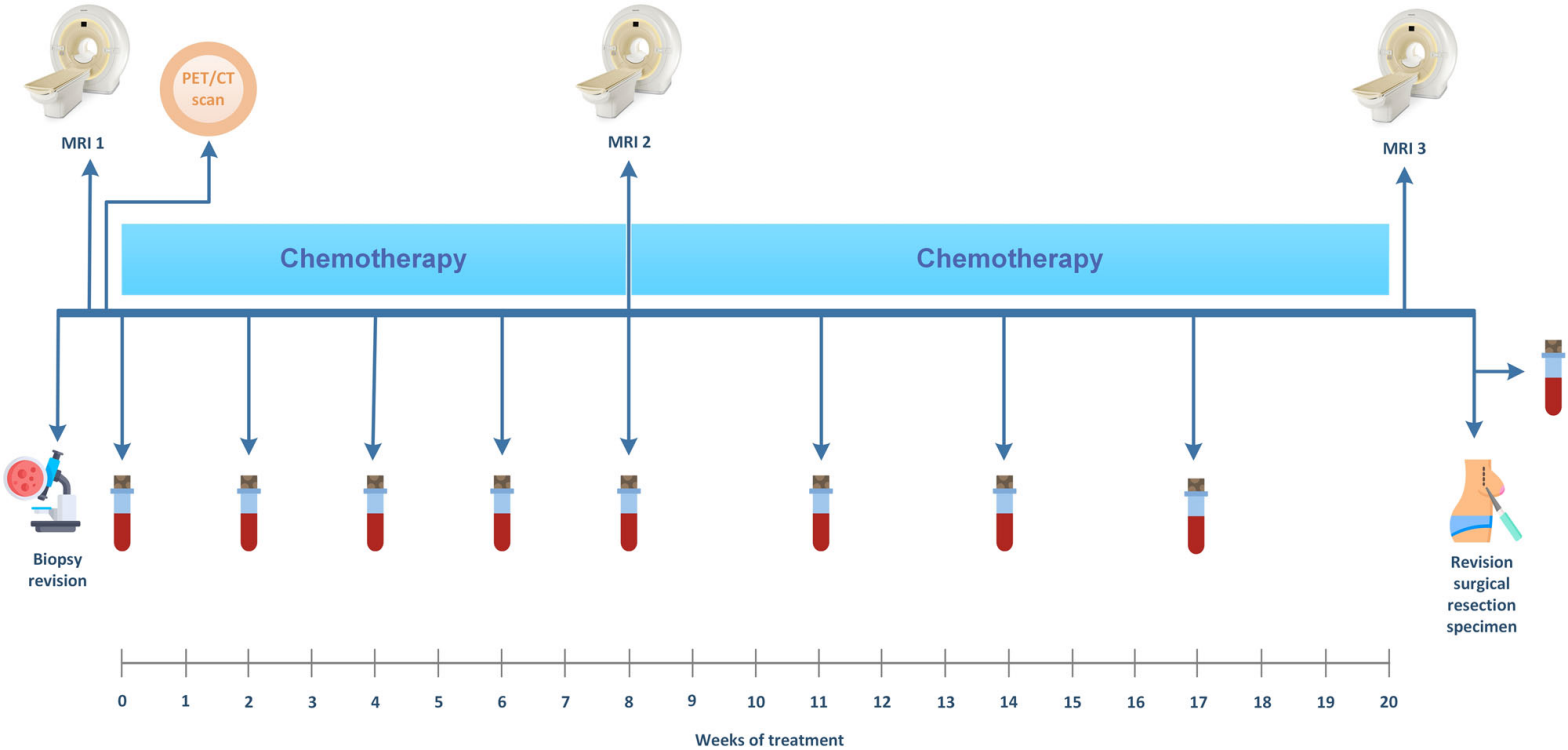
Schematic overview of the study procedures. All patients underwent a 3 T MRI of the breast and a whole body positron emission tomography/CT before treatment. MRI scans were also performed during and after treatment. Blood samples were collected before every chemotherapy cycle and before surgery. The exact moment of blood collection varied depending on the treatment schedule as described in the methods. This image has been designed using images from Flaticon.com.

An overview of the study procedures is shown in Fig. 2. All patients had a PET/CT scan before start of NAC to exclude distant metastases. An experienced breast pathologist (PvD) who was unaware of non-pathologic predictors of response conducted a central revision of the diagnostic biopsy and surgical specimen. RCB^29^, the primary outcome measure, was determined according to the guidelines using the calculator provided by the MD Anderson website^30^. pCR was defined as RCB = 0. For MRI revision and liquid biopsy assessment, blinding to the outcome and predictors was maintained.

### 3T-MRI acquisition and analysis

MRI of the breast was performed pre-treatment, on-treatment and post-treatment (before surgery). MR imaging was performed on 3 T scanners (Philips Achieva, Ingenia or Ingenia Elition X or Siemens MAGNETOM Avanto, Spectra, Skyra or Vida) with dedicated double breast coils. A multi-parametric MRI protocol consisting of four sequences was used: (1) T2-weighted sequence, (2) High-Temporal-Resolution Dynamic Contrast-Enhanced MRI (DCE-MRI) sequence, (3) High-Spatial-Resolution DCE-MRI sequence, (4) diffusion-weighted imaging sequence. Central revision of MRI scans with assessment of tumor size (i.e., the largest diameter among sagittal, coronal and transverse view planes in mm) was performed by an experienced breast radiologist (EW).

For automated analysis of the MRI series, tumors were first automatically segmented using the method described by ref. ^31^. A set of multiparametric MRI features previously used for computer-aided diagnosis by ref. ^32^ were then extracted. One hundred one features were calculated for each lesion. These features included the total number of lesions, the absence/presence of enhancing lesions, 27 features were related to T2 intensity, 4 features described contrast-uptake kinetics on the perfusion sequence, 6 features were related to diameter, 9 to volume, 4 described the shape, 8 were related to heterogeneity of contrast uptake, 15 described slow contrast dynamics, 13 described the margin, and 13 described the apparent diffusion coefficient (ADC) values. If more than one lesion was present in the breast, the mean and standard deviation of the feature values across the lesions were used in the case of volumetric, diameter, shape and margin features, while for T2-weighted, perfusion kinetics and ADC features, the feature described the whole segmented region as one.

### Liquid biopsy collection and cell-free DNA extraction

Blood samples for liquid biopsy assessment were collected in Streck Cell-Free DNA BCT® tubes before every chemotherapy cycle, and after completion of NAC prior to surgery. For patients treated with AC-P this meant a blood sample was taken before every AC cycle and before the first, fourth, seventh and tenth weekly paclitaxel cycle and before surgery. For patients treated with PTCP, a blood sample was taken before every carboplatin cycle. For patients treated with 12 times weekly trastuzumab and paclitaxel, a blood sample was taken every 2 weeks. Within 1–5 days after blood collection, plasma was isolated after centrifuging whole blood at 1600 × *g* for 10 min. Plasma was stored at −80 °C until further processing. All technicians were blinded to primary and secondary outcome measures, as well as predictors.

**Fig. 3:**
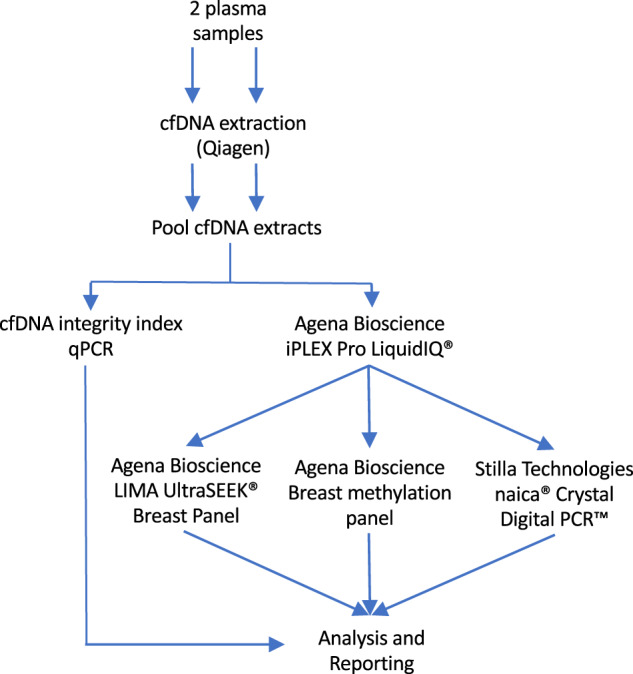
Overview of workflow blood samples after second centrifuging step. cfDNA was extracted from 2 plasma samples, after with the extracts were pooled. The extract was split, one part used for determining the cfDNA integrity index and the other for the LiquidIQ® Panel, followed by the mutation and methylation analysis.

Plasma samples were visually inspected for hemolysis, samples with severe hemolysis were excluded from further processing. Samples were centrifuged a second time for 10 min at 16,000 × *g* at 4 °C. cfDNA was isolated from 1–5 ml plasma using the QIAamp Circulating Nucleic Acids Kit (Qiagen GmbH), eluted in 50 µl AVE buffer (Qiagen GmbH). The eluates of matching plasma samples were pooled and stored in DNA LoBind tubes (Eppendorf AG) at −20 °C, resulting in 100 µl for downstream analysis. The extracted cfDNA was then assessed for quality and quantity as well as used for mutation and methylation analysis (Fig. 3).

The quality and quantity of each cfDNA sample was assessed in duplicate using 1.5 µL extracted cfDNA with the iPLEX Pro LiquidIQ® Panel (Agena Bioscience, San Diego, USA) with the MassARRAY® System (Agena Bioscience) as described previously^33^. Briefly, this returns the number of amplifiable cfDNA copies, detects long DNA fragments (>340 bp) originating from cell necrosis, and an estimate of the level of white blood cell (WBC) contamination. cfDNA fragmentation was assessed with cfDNA integrity index qPCR. This index is based on the ratio of long fragmented DNA (assay 1) and short fragmented DNA (assay 2), thereby indicating potential contamination of the DNA sample with genomic DNA (long fragments)^34^.

For quality evaluation and decisions about sample exclusion, the results of the iPLEX Pro LiquidIQ® Panel were decisive. Samples with amplifiable copy number representing <2 ng/max. assay volume were excluded from both UltraSEEK® Breast Panel (Agena Bioscience, San Diego, USA) and the Breast Methylation Panel (Agena Bioscience). Samples with a WBC contamination >50% and >75% were excluded from UltraSEEK® Breast Panel and the Breast Methylation Panel, respectively.

### Detection of somatic mutations

Somatic mutations were detected using the UltraSEEK® panel on the MassARRAY® (Agena Bioscience, San Diego, USA), which was previously described and evaluated^35–38^. The core UltraSEEK® Breast Panel v1.0, as described previously^39^, was extended with the Custom GATA3 and FOXA1 Panel (Table 3). Proprietary reagents were used. Starting from two global multiplex polymerase chain reactions (PCR), the panel tests 52 mutations across 7 oncogenes (core: AKT1, ERBB2, ESR1, PIK3CA, and TP53; extended: FOXA1, GATA3) in 12 multiplex assays. PCR was performed using on average 13 ng of cfDNA according to the manufacturer’s instructions. Amplified products were treated with shrimp alkaline phosphatase (SAP) and the PCR/SAP product was aliquoted in a new 96-well plate for downstream extension and termination reaction according to the manufacturer’s instructions. The single-base extended oligonucleotides were captured by streptavidin-coated magnetic beads and biotin-labeled following manufacturer’s instructions. The products were then transferred to the MassARRAY System with Chip Prep Module 96 (CPM96) that automatically performs desalting (resin), transfer of analyte and calibrant to the SpectroCHIP® Arrays and loading of SpectroCHIP® Arrays. Data were automatically acquired via matrix-assisted laser desorption/ionization time-of-flight (MALDI-TOF) mass spectrometry using the MassARRAY Analyzer. Data analysis was performed using Typer Analyzer software version 5.0.6 and the Somatic Variant Report version 1.0 (Agena Bioscience). Variant allele frequency was calculated for the signal intensity of the mutant allele, which had been normalized against the 5 capture control peaks found in the spectrum and an assay specific correction coefficient. The capture control peaks are biotin-labeled, nonreactive oligos, which are added to the extension reaction and used as an internal control for the streptavidin-bead capture and elution of the mutant extension product steps. In preparatory work, the analytical sensitivity of this panel (defined as the minimum percentage of mutant allele frequency in reference material which was measured as positive with probability ≥90%) was 100% at 1% mutation allele frequency (MAF) and 44% at 0.2% MAF. Additionally, HER2 amplification and PIK3CA hotspot mutations (p.E542K (c.1624G > A), p.E545K (c.1633G > A), p.H1047R (c.3140 A > G)) were evaluated by six-color Crystal Digital PCR™ with Sapphire Chips on the naica® system (Stilla Technologies, Villejuif, France), using sets of primers, TaqMan® hydrolysis probes (Table 4) (Eurogentec, Seraing, Belgique) and the naica® multiplex PCR MIX (Stilla Technologies, Villejuif, France). Detection of HER2 amplification is based on a ratio of ERBB2 and TSN concentrations, TSN being considered as a housekeeping reference gene. MRM1 is located on the same chromosome as ERBB2 and is used as a control for chr17 polysomy. The total DNA sample concentration is measured by calculating the mean of the three wild-type (WT) targets PIK3CA, MRM1 and TSN. The LIMA BC panel also enables quantification of a synthetic DNA (PhiX) PCR control added to the PCR after extraction but prior to amplification. The 6-color experiments were performed by Crystal Digital PCR™ with Sapphire Chips on the naica® system (Stilla Technologies, Villejuif, France). Samples were partitioned into 2D-droplets monolayers and thermocycled using the naica® Geode instrument. Cycling conditions were 95 °C for 3 min, followed by 50 cycles of 95 °C for 15 s and 62 °C for 30 s. Sapphire Chips were imaged on the naica® Prism6 instrument. Droplet identification and fluorescence measurements in each detection channel were performed using Crystal Reader and Crystal Miner software v3.0.6.2. After the automatic application of a spillover compensation matrix to the raw fluorescence data^40^, a threshold was applied to discriminate the positive droplets from the negative droplets, using the automated tool of the Crystal Miner software. This panel for the naica® system was designed and validated using reference materials purchased from commercial providers (WT human genomic DNA (ENZ-GEN117-0100, Enzo Life Sciences, Farmingdale New York USA), mutated synthetic DNA (Ultramer™ DNA oligonucleotides, IDT, Coralville Iowa USA) and PCR control PhiX DNA (phiX174 RF1 DNA SD0031, Thermo-Fischer, Waltham Massachusetts USA). Clinical validations were performed on cfDNA from healthy donors with the NucleoSnap cfDNA kit (740300.10, Macherey-Nagel, Dueren Germany). Technical performances were evaluated including the Limit of Blank (LoB), Limit of Detection (LoD), linearity and repeatability. LoB of each mutant target were measured on 30 cfDNA extracted from healthy donors and LoD of each mutant target were theoretically extrapolated from the LoB. LoB and LoD characterization method is described on the Stilla website^41^. The LoB and LoD for *PIK3CA* mutations in the LIMA BC panel were 0.07 and 0.16 copies/µL, respectively, corresponding to a LoB of 0.21 pg/µl and a LoD of 0.48 pg/µL. The control ratio (*ERBB2*/*TSN*) for *HER2* amplification was measured on 33 cfDNA from healthy donors. Linearity and sensitivity was assayed on six serial dilutions (three replicates per point) of mutated DNA, each bearing one of the following mutations: *PIK3CA* p.E542K (c.1624G > A), p.E545K (c.1633G > A), p.H1047R (c.3140 A > G). A clinical validation was performed on cfDNA extracted from breast cancer patients plasma. Data analysis was performed on 424 cfDNA samples with the naica® system and the digital PCR breast cancer panel. Each sample was analyzed with three technical replicates using on average 21.5 ng cfDNA in total, with inclusion of a WT control and a positive control for each run. After the Crystal Digital PCR™ and the imaging of the Sapphire Chips, an analysis template was used to automatically calculate the cfDNA concentrations and mutational status of each sample. For the quantification of *PIK3CA* mutations, the MAF (mutation allele frequency) was calculated by dividing the concentration of *PIK3CA* mutated DNA by the average of the three WT targets (*PIK3CA* WT, *TSN*, *MRM1*).

**Table 3:** LIMA UltraSEEK Breast Panel variant list.

Gene	Coverage	Panel	# of Variants
AKT1	E17K, L52R	Core	2
ERBB2 (HER2)	G309A, G309E, S310F, L755R, L755S, L755_T759del, D769H, D769Y, V777L, L869R	Core	10
ESR1	A283V, K303R, E380Q, V392I, S463P, V534E, L536R, L536Q, Y537N, Y537S, Y537C, D538G, S576L	Core	13
FOXA1	E24K, I176M, I176V, D226N, S250F, F266L	Extended	6
GATA3	S93F, S137L, M294K, D336fs17, R365G, P409fs37	Extended	6
PIK3CA	N345K, C420R, E542K, E545A, E545K, E545Q, H1047L, H1047R	Core	8
TP53	R175H, R213*, Y220C, R248W, R248Q, R273C, R273H	Core	7
Total Variants			52

**Table 4:** Breast cancer panel oligonucleotides description.

Oligo name	Type	5′ to 3′ Sequence	Modifications (5′ - 3′)
ERBB2 F	Primer	ACG-GAC-GTG-GGA-TCC-TGC-A	/
ERBB2 R	Primer	CTT-CTC-ACA-CCG-CTG-TGT-TCC-AT	/
ERBB2 Probe	Taq Probe	ACA-ACC-AAG-AGG-TGA-CAG-CAG-A	6-FAM - BHQ1
PIK3CA H1047 F	Primer	GCT-TTG-GAG-TAT-TTC-ATG-AAA-CA	/
PIK3CA H1047 R	Primer	AGA-TCC-AAT-CCA-TTT-TTG-TTG-TC	/
PIK3CA H1047 WT Probe	Taq Probe	CCA-CCA-TGA-TGT-GCA-T	YY - MGB Eclipse
PIK3CA H1047R Probe	Taq Probe	CAC-CAT-GAC-GTG-CAT	ROX - MGB Eclipse
PIK3CA E542E545 F	Primer	CTC-AAA-GCA-ATT-TCT-ACA-CGA-G	/
PIK3CA E542E545 R	Primer	TTA-CCT-GTG-ACT-CCA-TAG-AAA-ATC	/
PIK3CA E542K Probe	Taq Probe	CCT-CTC-TCT-AAA-ATC-ACT-G	ROX - MGB Eclipse
PIK3CA E545K Probe	Taq Probe	TTC-TCC-TGC-TTA-GTG-ATT-T	ROX - MGB Eclipse
MRM1 F	Primer	GTG-GAT-AAG-GTC-ATC-ACC-A	/
MRM1 R	Primer	CAA-GGT-GCT-TAG-GAA-CTC-G	/
MRM1 Probe	Taq Probe	ACG-TCC-CTC-ATT-CTC-TAT-GTG-CC	Cy3 - BHQ2
TSN F	Primer	CAG-CGT-GAC-TGC-TGG-AGA-CTA-CT	/
TSN R	Primer	ACC-GGA-ATC-CAG-CTC-ATT-GAT	/
TSN Probe	Taq Probe	ACC-CCT-CCA-CAT-CTC-CAC-CTT	Cy5 - BHQ3
PhiX174 F	Primer	TCT-TTC-CAA-GCA-ACA-GCA-G	/
PhiX174 R	Primer	AAT-ACT-GAC-CAG-CCG-TTT-GA	/
PhiX174 Probe	Taq Probe	TCC-GAG-ATT-ATG-CGC-CAA-ATG-C	Atto700 - BHQ3

### Methylation

For the detection of methylation, a custom 14-gene Breast Methylation Panel v1.0 and proprietary reagents (Agena Bioscience) were used (Table 5). The assay, of which the workflow has been described previously^42^, uses methylation-sensitive restriction enzymes to eliminate the non-tumor, unmethylated fraction of the DNA. The panel contained assays for digestion quality control and total cfDNA quantification to enable downstream data analysis. 2–15 ng of cfDNA were used in the digestion reaction. PCR was performed according to the manufacturer’s instructions (Agena Bioscience, San Diego, USA). The undigested methylated ctDNA fraction was co-amplified in the presence of a synthetic oligonucleotide to permit competitive PCR amplification. PCR products were treated with protease enzyme and aliquots were transferred in a new 96-well plate for treatment with shrimp alkaline phosphatase (SAP) and downstream single base extension and termination reaction according to the manufacturers instructions. The products were transferred to and analyzed on the MassARRAY® System as described above.

**Table 5:** Marker list methylation panel.

Gene	Genomic Location	Reference
*AKR1B1*	chr7:134459123	Fackler^19^
*APC*	chr5:112737754	Radpour^47^
*ARHGEF7*	chr13:111115541	Fackler^19^
*BRCA1*	chr17:43125416	Radpour^47^
*COL6A2*	chr21:46098888	Fackler^19^
*GPX7*	chr14:37592244	Fackler^19^
*HIST1H3C*	chr1:52602513	Fackler^19^
*MDGI*	chr17:48578124	Liggett^48^
*RASGRF2*	chr1:31373414	Fackler^19^
*RASSF1A*	chr5:80960894	Fackler^19^
*TM6SF1*	chr3:50340798	Fackler^19^
*FOXA1*	chr5:180591531	Nunes^49^
*SCGB3A1*	chr15:83107646	Nunes^49^
*TMEFF2*	chr2:192194694	Fackler^19^

Data analysis was performed using an MS Excel macro-based analysis tool to normalize signal and calculate a methylation score per sample. Normalization is performed to make the detected methylation levels comparable between samples.

Based on the methylation signal assessed for the six genes with highest significance (AKR1B1, GPX7, HIST1H3C, SCGB3A1, TM6SF1, TMEFF2) a methylation score per sample was calculated. The methylation score is the sum of the methylation copies (normalized to 10 ng DNA input) for the six genes listed above. A positive methylation score is considered for z-score ≥3 with the z-score being calculated as the average methylation score of a normal sample cohort divided by the standard deviation for that cohort. Methylation score values below the cutoff were set to the cutoff divided by two to reduce noise.

### Statistical analysis

Statistical analysis consisted of data pre-processing (i.e, transformations, dimensionality reduction by principle component analysis (PCA) of specific feature-sets (e.g., T2 MRI features), and single imputation of missing values) followed by model development and model evaluation using internal cross-validation. The model development steps were incorporated in the cross-validation procedure. By contrast, the pre-processing steps were not incorporated in the cross-validation, in part because these were deemed to have at most minor potential to increase overfitting. The data pre-processing steps were unsupervised, ultimately resulting in a complete data set of reduced dimensionality compared to the original dataset. First, liquid biopsy and MRI-based (continuous) variables were transformed—using a log transformation and Box-Cox procedure, respectively—into variables with normal-shaped distributions. Second, to accommodate for incomplete patient data in the model development and evaluation, we imputed missing values. Specifically, each missing value of a variable with repeated measurements was imputed using linear interpolation between the last and next observations. In the absence of either a last or a next observation, the missing value was imputed with the next or last observation, respectively. If neither a last nor next observation was available, we used the mean value across patients. Third, the liquid biopsy variables with repeated measurements were aggregated into nine bins corresponding with the fraction of chemotherapy that was completed at the measurement time. The value associated with the first bin was defined by this fraction being zero (i.e., start of chemotherapy); the other bins were formed by dividing the interval (0,1] into eight intervals of the same length. Measurements associated with each bin were averaged per variable and per patient, yielding bin averages for all downstream analyses instead of the original liquid biopsy variables. Fourth, liquid biopsy and MRI variables were standardized. In the fifth step, we applied principal component (PC) dimensionality reduction to each liquid biopsy or MRI “feature set” (groups of variables describing similar information of a patient describing similar information of a patient). The dimensionality reduction was accomplished by transforming the feature set variables into equally many new variables, the principal components, with progressively smaller variance. The first principal component is defined as a linear combination of the variables with the greatest variance among all linear combinations whose squared coefficients sum to 1. The definition of every subsequent principal component is the same except that we additionally require the linear combinations to be linearly independent of all previous components. For each feature set, we selected the minimum number of principal components that together accounted for at least 80% of the total variance. For the PC dimensionality reduction step, repeated measurements of (i.e., time-specific versions of) the same variables were treated as distinct variables to account for possible clustering or time trends within individuals. In other words, PC dimensionality reduction was applied to the data in “wide format”.

We developed three types of models: feature set-only models, each developed with the variables of one feature set as predictor variables; ensemble models, which combine the predictions of all clinical, liquid biopsy or MRI feature set-only models; and ensemble-of-ensembles models, which combine the predictions of different ensemble models (Table 6). Each of these models was derived from predictor information that was available pre-treatment, on-treatment and post-treatment. Predictors that had been previously established in the literature and were part of the standard diagnostic workflow were used to build the pre-treatment clinical-radiological prediction model. This model consisted of tumor grade, nodal status, tumor size on baseline MRI, and receptor subtype (ER−/HER2−, ER + −/HER2+ or ER + /HER2−)^43–45^. For the on-treatment and post-treatment models, the relative change in tumor size on MRI compared to that on baseline MRI as measured by the radiologist was added. A clinical-only model was also developed without tumor size on MRI.

**Table 6:** Prediction models that were developed with their candidate predictors.

Model name	Candidate predictors used to develop model
Clinical-radiological	Ensemble of tumor grade, nodal status, receptor subtype, radiologist-assessed tumor size on baseline MRI (+/−relative change in tumor size for non-baseline time-points)
Liquid biopsies	Ensemble of cfDNA load, cfDNA integrity, mutation and methylation feature sets
MRI features	Ensemble of diameter, heterogeneity, kinetics, margin, shape T2, uptake and volume feature sets
Clinical-radiological + liquid biopsies	Ensemble of clinical-radiological and liquid biopsy ensembles
Clinical-radiological + MRI features	Ensemble of clinical-radiological and MRI features ensembles
Liquid biopsies + MRI features	Ensemble of liquid biopsy and MRI features ensembles
Clinical-radiological + Liquid biopsies + MRI features	Ensemble of clinical-radiological, liquid biopsy and MRI features ensembles
Only clinical	Ensemble of tumor grade, nodal status, receptor subtype
Only clinical + liquid biopsies	Ensemble of only clinical and liquid biopsy ensembles
Only clinical + MRI features	Ensemble of only clinical and MRI features ensembles
Only clinical + MRI features + liquid biopsies	Ensemble of only clinical, liquid biopsy and MRI features ensembles.

Each model was a (main effects) linear regression model with RCB as the dependent variable, fit using LASSO with the penalty parameter set at the value that yielded the lowest mean squared error in an inner-loop LOOCV scheme. To estimate the expected out-of-sample performance of the various models in terms of discrimination, we used an additional outer-loop LOOCV, applying all model development steps to the training data. Discrimination was evaluated using ROC curves and, in particular, AUC. Presented confidence intervals are 95% pointwise confidence intervals constructed using a percentile bootstrap approach applied directly to the pairs of RCB values and leave-one-out predictions. Because these confidence intervals do not capture the variability in model parameters across datasets means that they should be interpreted with extra caution. How to estimate accurate CIs in studies like this is an active area of investigation^46^. We recommend that the reported CIs are used at most to guide the generation of new hypothesis rather than to reject hypotheses. The estimation of p-values is similarly problematic, even further augmented by multiple testing issues, which is why we refrain from reporting these in this exploratory study.

Each patient was considered one case. One patient with a bilateral tumor was considered one case in which the radiological tumor size and computer extracted diameter were taken as the sum of both tumors. In all other computer extracted MRI features the mean of both tumors was taken. Receptor subtype and grade were the same for both tumors. All statistical analysis were performed in R software version 4.2.2.

### Reporting summary

Further information on research design is available in the Nature Research Reporting Summary linked to this article.

### Supplementary information

Supplementary Information

Reporting summary

## Data Availability

Data generated or analyzed during the study are available from the corresponding author by request.

## Code availability

R code used for statistical analysis is available from the corresponding author by request.
