# Supplementary material for: Predicting response to neoadjuvant chemotherapy with liquid biopsies and multiparametric MRI in patients with breast cancer
Source: NPJ Breast Cancer. 2024 Jan 20;10:10. doi: 10.1038/s41523-024-00611-z (PMC10799888; doi:10.1038/s41523-024-00611-z)
Supplement: Supplementary file 1 — Supplementary Information [file 41523_2024_611_MOESM1_ESM.pdf]

Supplementary figure 1

Plot on missing liquid biopsy samples. Each horizontal row represent a missing missing data pattern and how many times it occured. Each vertical row represents a timepointat which a liquid biopsy was collected. A blue square means the sample was avaiable for analysis, a red square means it was missing.

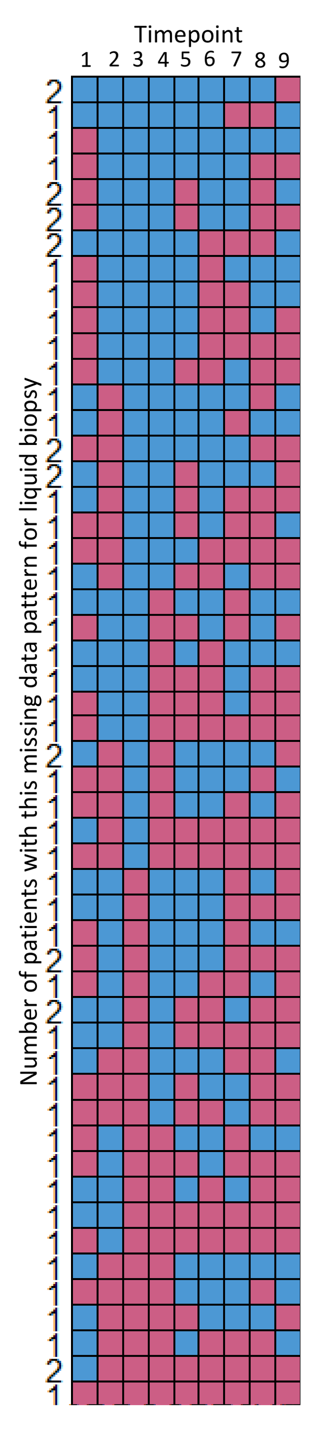

Supplementary figure 2

ROC curves of models at timepoint 1 (a), timepoint 2 (b, d-i) and timepoint 3 (c, j-o) discriminating between pCR (RCB= 0) and residual disease (RCB>0). Sensitivity = the % of patients with pCR correctly classified by the model. Specificity = the % of patients with residual disease correctly classified by the model

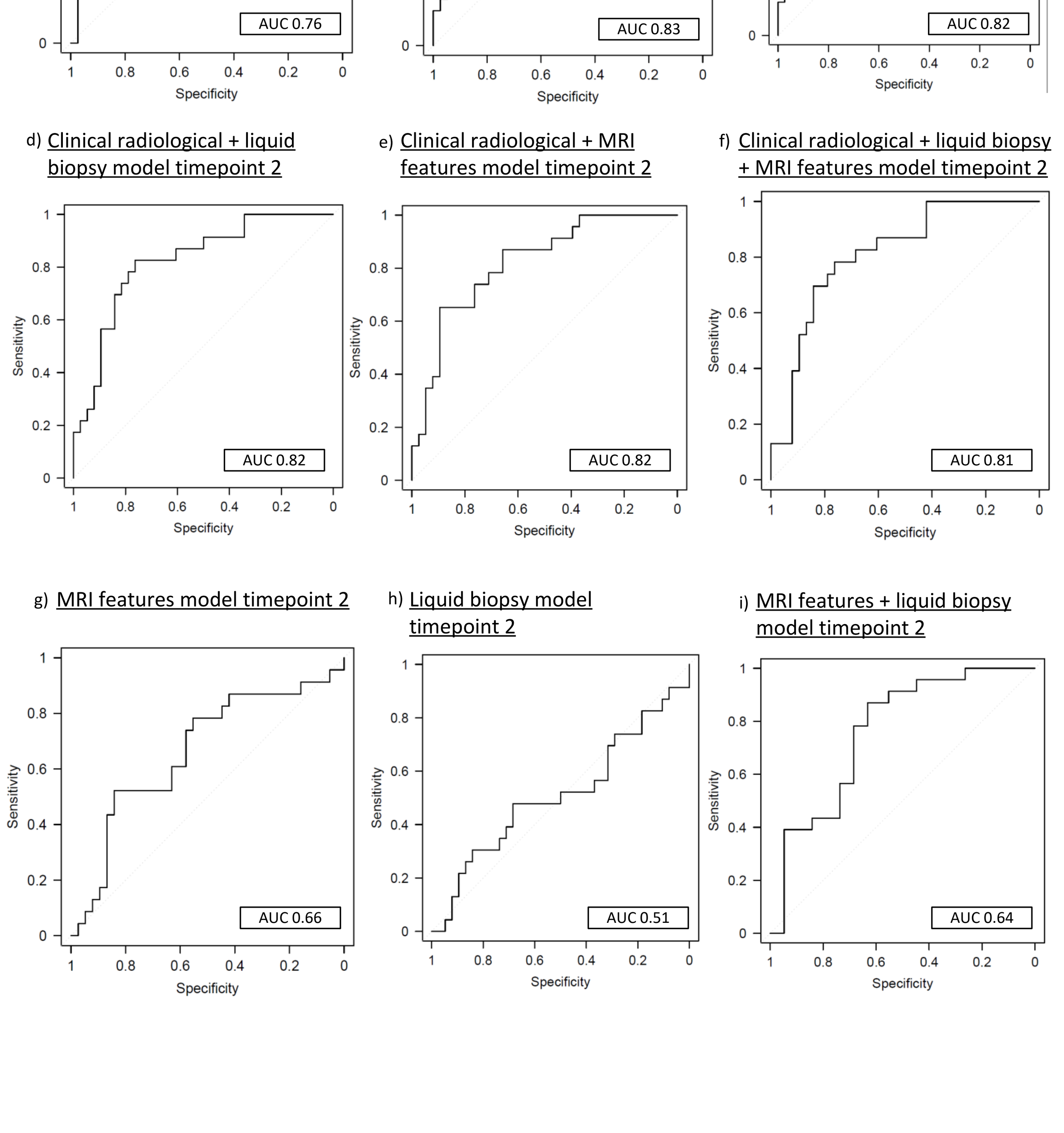

Supplementary figure 2 (continued)

ROC curves of models at timepoint 1 (a), timepoint 2 (b, d-i) and timepoint 3 (c, j-o) discriminating between pCR (RCB= 0) and residual disease (RCB>0). Sensitivity = the % of patients with pCR correctly classified by the model. Specificity = the % of patients with residual disease correctly classified by the model

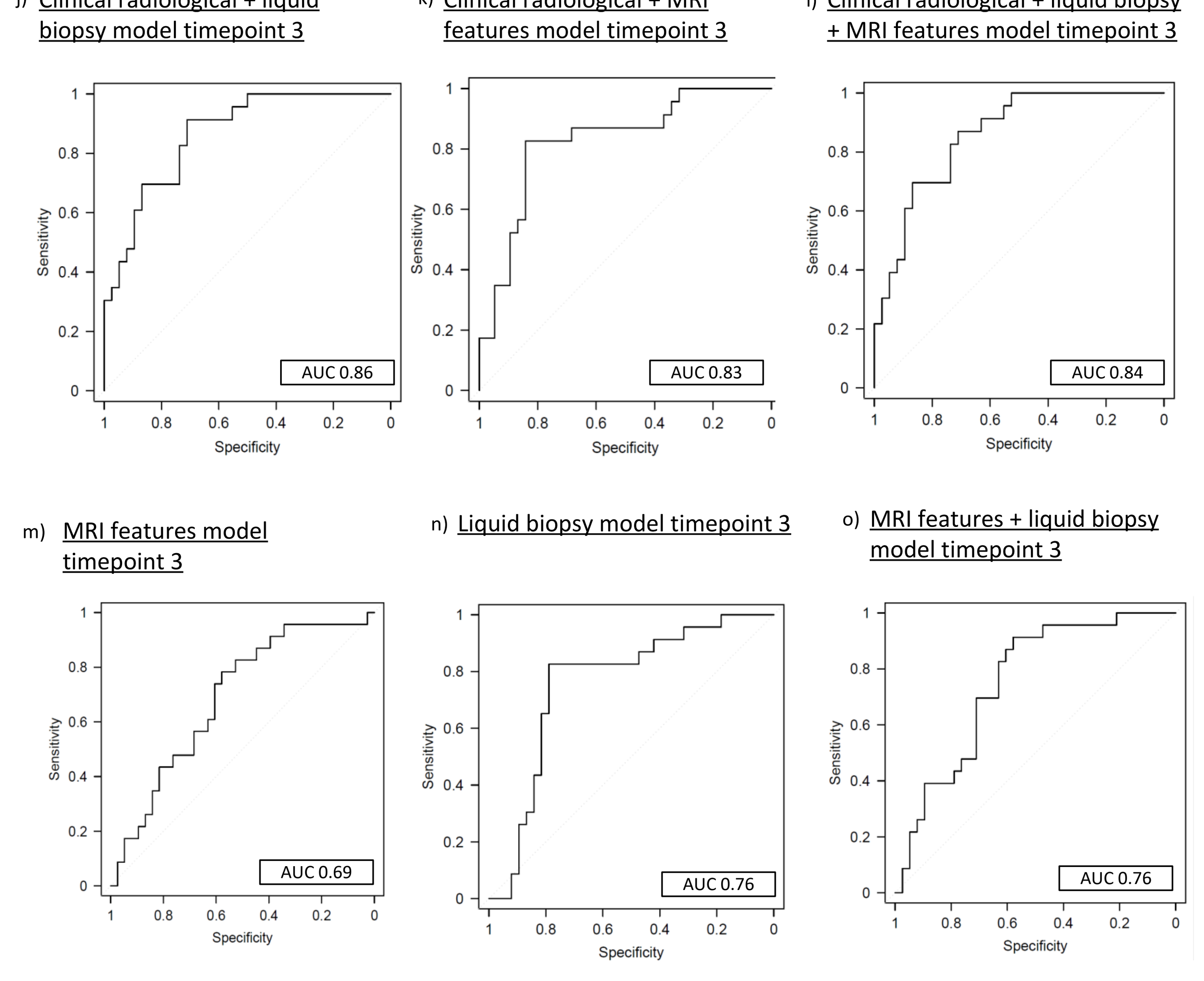

Supplementary figure 3

Calibration plots of models at timepoint 1 (a), timepoint 2 (b, d-i) and timepoint 3 (c, j-o)

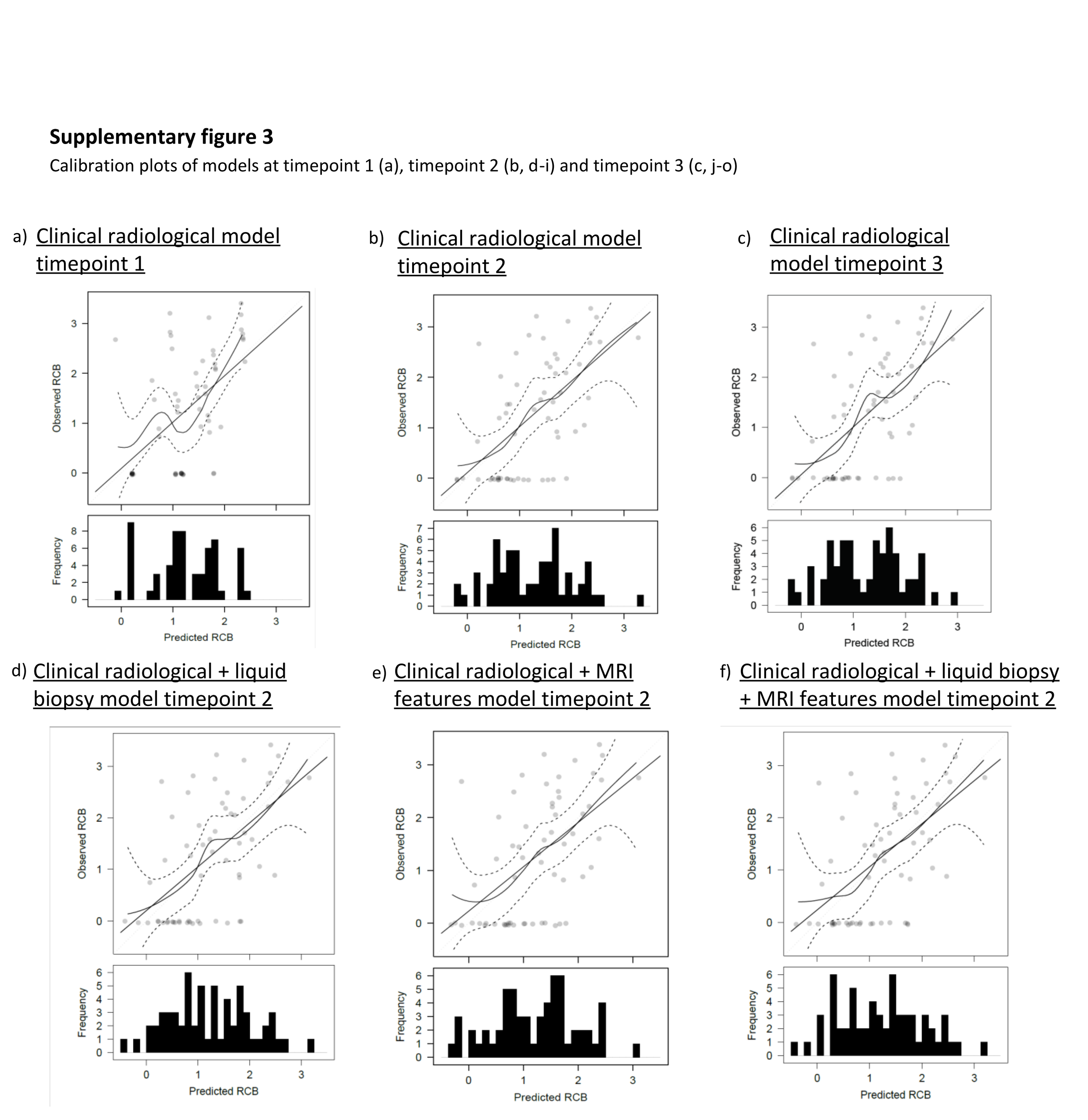

Supplementary figure 3 (continued)

Calibration plots of models at timepoint 1 (a), timepoint 2 (b, d-i) and timepoint 3 (c, j-o)

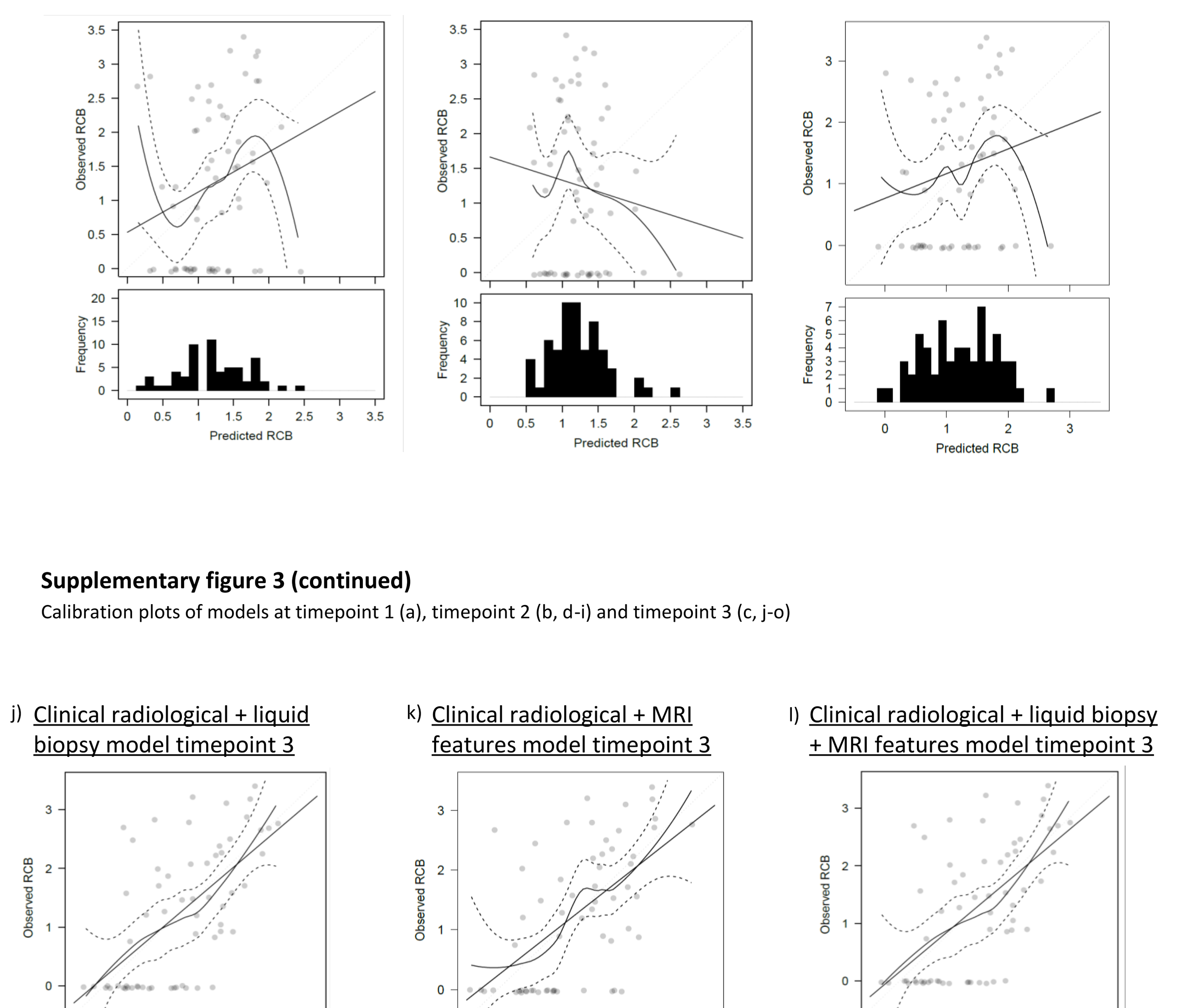

Supplementary figure 4

Scatterplots depicting the correlation including Pearson correlation coefficient between RCB and the trend of biomarkers between timepoint 1 and 3 for the methylation markers (a-9) and the total amount of ctDNA (h)

a) methylation index score b) AKR1B1 c) GPX7 d) HIST1H3C e) SCGB3A1 f) TM6SF1 g) TMEFF2 and h) total cfDNA

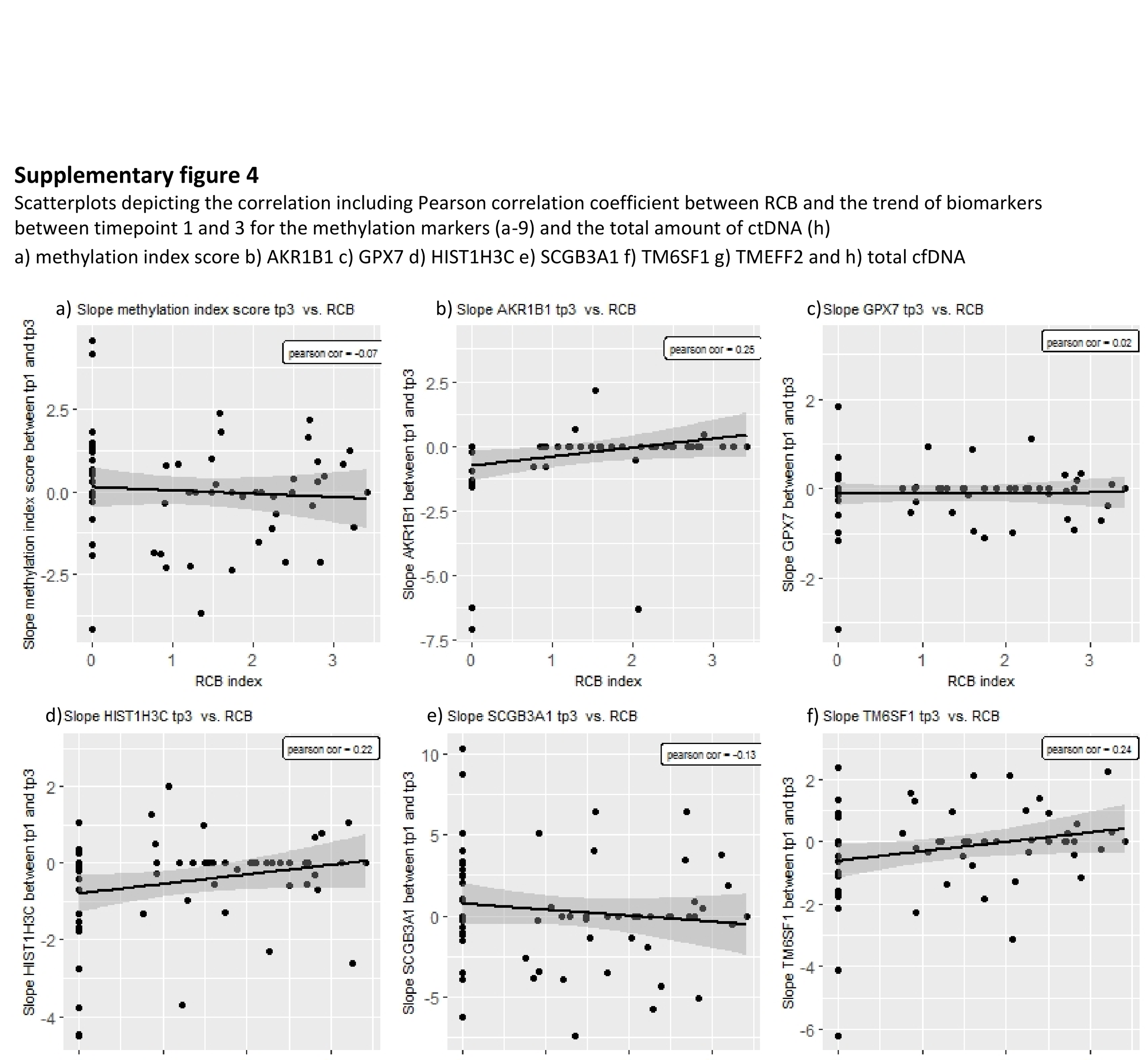

Supplementary Table 1: ctDNA alterations detected

Alteration*	Number of times it was detected**
ESR1:pV392I	1
PIK3CA:pE542K	1
PIK3CA:pE545K	2
TP53:pR273H	2
TP53:pY220C	3
TP53:pR175H	1
ERBB2 amplification***	5

*The PIK3CA mutations were detected by both the UltraSEEK and naica Crystal Digital PCR technologies
**None of the alterations were detected in more than one sample of the same patient
*** ERBB2 amplification was not found in all patients in HER2+ disease on IHC

Supplementary Table 2: Summary of model performance corrected for optimism by leave one out cross validation including naive confidence intervals.

Model	Pre-treatment		On-treatment		Post-treatment	
	**AUC** *(95% CI)*	**MSE** *(95% CI)*	**AUC** *(95% CI)*	**MSE** *(95% CI)*	**AUC** *(95% CI)*	**MSE** *(95% CI)*
**Clinical-radiological**	0.76 *(0.62-0.88)*	0.94 *(0.63-1.33)*	0.83 *(0.71-0.93)*	0.86 *(0.60-1.16)*	0.82 *(0.71-0.92)*	0.87 *(0.61-1.18)*
**Liquid biopsies**	NA	NA	0.51 *(0.34-0.67)*	1.57 *(1.22-1.98)*	0.76 *(0.63-0.88)*	1.27 *(1.00-1.60)*
**MRI features**	NA	NA	0.66 *(0.51-0.81)*	1.27 *(0.92-1.66)*	0.69 *(0.53-0.81)*	1.25 *(0.93-1.60)*
**Clinical-radiological + liquid biopsies**	0.77 *(0.64-0.88)*	0.99 *(0.63-1.42)*	0.82 *(0.71-0.92)*	0.88 *(0.58-1.18)*	0.86 *(0.76-0.94)*	0.82 *(0.54-1.12)*
**Clinical-radiological + MRI features**	0.77 *(0.64-0.88)*	0.99 *(0.65-1.41)*	0.82 *(0.70-0.92)*	0.88 *(0.57-1.23)*	0.83 *(0.70-0.93)*	0.89 *(0.56-1.24)*
**Liquid biopsies + MRI features**	NA	NA	0.64 *(0.47-0.78)*	1.38 *(1.01-1.83)*	0.76 *(0.64-0.88)*	1.24 *(0.92-1.57)*
**Clinical-radiological + Liquid biopsies + MRI features**	0.76 *(0.63-0.88)*	1.03 *(0.67-1.46)*	0.81 *(0.70-0.91)*	0.91 *(0.59-1.25)*	0.86 *(0.75-0.94)*	0.84 *(0.56-1.16)*
**Only clinical**	0.76 *(0.62-0.87)*	0.94 *(0.62-1.36)*	0.76 *(0.62-0.87)*	0.94 *(0.62-1.36)*	0.76 *(0.62-0.87)*	0.94 *(0.62-1.36)*
**Only clinical + liquid biopsies**	0.76 *(0.63-0.87)*	0.98 *(0.62, 1.42)*	0.78 (0.65-0.88)	0.98 (0.67-1.39)	0.85 (0.74-0.94)	0.89 (0.58-1.29)
**Only clinical + MRI features**	0.76 *(0.63-0.87)*	0.98 *(0.63-1.39)*	0.82 (0.71-0.91)	0.92 (0.57- 1.41)	0.84 (0.72-0.93)	0.95 (0.59- 1.42)
**Only clinical + MRI features + liquid biopsies**	0.76 *(0.63-0.87)*	1.01 *(0.65-1.43)*	0.81 *(0.70-0.91)*	0.97 *(0.62-1.45)*	0.85 *(0.74-0.93)*	0.91 *(0.57-1.35)*

CI = confidence interval, AUC = area under the receiver operating characteristic curve for predicting pCR (RCB 0), MSE = mean standard error, measures the average squared difference between the observed and predicted values. A lower value indicates a better fit of the model. NA = not applicable, no predictors remained in the model.

Supplementary Table 3: Coefficients of prediction models

Model	Predictor	Coefficient
**Clinical-radiological pre-treatment**	(Intercept)	1.50
	IHC subtype ER-/HER2-	-0.76
	IHC subtype ER+-/HER2+	-0.74
	Grade 3	-0.54
	Tumor size on baseline MRI	0
	Nodal status	0.89
	(Intercept)	0.84

**Clinical-radiological on-treatment**	IHC subtype ER-/HER2-	-0.56
	IHC subtype ER+-/HER2+	-0.58
	Grade 3	-0.45
	Tumor size on baseline MRI	0
	Nodal status	0.71
	Relative change in tumor size on MRI 2 compared to baseline	0.01
**Clinical-radiological post-treatment**	(Intercept)	0.83
	IHC subtype ER-/HER2-	-0.54
	IHC subtype ER+-/HER2+	-0.55
	Grade 3	-0.45
	Tumor size on baseline MRI	0
	Nodal status	0.70
	Relative change in tumor size on MRI 2 compared to baseline	0.01
	Relative change in tumor size on MRI 3 compared to baseline	0
**Liquid biopsiesies pre-treatment**	(Intercept)	1.25
	Amplifiable copies	0
	cfDNA integrity	0
	Methylation scores	0
	Mutations	0
	Plasma DNA concentration	0
**MRI features pre-treatment**	(Intercept)	1.25
	ADC	0
	Diameter	0
	Heterogeneity	0
	Kinetics	0
	Margin	0
	Shape	0
	T2	0
	Uptake	0
	Volume	0
**Liquid biopsies on-treatment**	(Intercept)	0
	Amplifiable copies	1.00
	cfDNA integrity	0
	Methylation scores	0
	Mutations	0
	Plasma DNA concentration	0
**MRI features on-treatment**	(Intercept)	0.12
	ADC	0
	Diameter	0

	Heterogeneity	0
	Kinetics	0
	Margin	0
	Shape	0
	T2	0
	Uptake	0
	Volume	0.90
**Liquid biopsies post-treatment**	(Intercept)	-2.82
	Amplifiable copies	1.15
	cfDNA integrity	0
	Methylation	2.10
	Mutations	0
	Plasma DNA concentration	0
**MRI features post-treatment**	(Intercept)	-0.33
	ADC	0
	Diameter	0.10
	Heterogeneity	0
	Kinetics	0
	Margin	0
	Shape	0
	T2	0.39
	Uptake	0
	Volume	0.78
**Clinical-radiological + liquid biopsies pre-treatment**	(Intercept)	-0.15
	Clinical-radiological model	1.12
	Liquid biopsies model	0
**Clinical radiological + MRI features pre-treatment**	(Intercept)	-0.15
	Clinical-radiological model	1.12
	Computer extracted MRI features model	0
**Clinical radiological + LB + MRI features pre-treatment**	(Intercept)	-0.15
	Clinical-radiological model	1.12
	Liquid biopsies model	0
	Computer extracted MRI features model	0
**Clinical radiological + liquid biopsies on-treatment**	(Intercept)	-0.65
	Clinical-radiological model	1.08
	Liquid biopsies model	0.44
**Clinical radiological + MRI features on-treatment**	(Intercept)	-0.50
	Clinical-radiological model	1.02
	Computer extracted MRI features model	0.38

**Liquid biopsies + MRI features on-treatment**	(Intercept)	-0.91
	Liquid biopsies model	0.62
	Computer extracted MRI features model	1.11
**Clinical radiological + liquid biopsies + MRI features on-treatment**	(Intercept)	-0.86
	Clinical-radiological model	0.98
	Liquid biopsies model	0.39
	Computer extracted MRI features model	0.32
**Clinical radiological + liquid biopsies post-treatment**	(Intercept)	-0.67
	Clinical-radiological model	0.98
	Liquid biopsies model	0.55
**Clinical radiological + MRI features post-treatment**	(Intercept)	-0.45
	Clinical-radiological model	1.03
	Computer extracted MRI features model	0.33
**Liquid biopsies + MRI features post-treatment**	(Intercept)	-0.47
	Liquid biopsies model	0.67
	Computer extracted MRI features model	0.71
**Clinical radiological + liquid biopsies + MRI features post-treatment**	(Intercept)	-0.55
	Clinical-radiological model	0.94
	Liquid biopsies model	0.50
	Computer extracted MRI features model	0
**Only clinical model**	(Intercept)	1.55
	IHC subtype ER-/HER2-	-0.85
	IHC subtype ER+-/HER2+	-0.88
	Grade 3	-0.59
	Nodal status	0.96
**Only clinical + liquid biopsies pre-treatment**	(Intercept)	-0.02
	Only clinical model	1.02
	Liquid biopsies model	0
**Only clinical + MRI features pre-treatment**	(Intercept)	-0.02
	Only clinical model	1.02
	Computer extracted MRI features model	0
**Only clinical + MRI features + liquid biopsies pre-treatment**	(Intercept)	-0.02
	Only clinical model	1.02
	Liquid biopsies model	0
	Computer extracted MRI features model	0
**Only clinical + liquid biopsies on-treatment**	(Intercept)	-0.61
	Only clinical model	0.93
	Liquid biopsies model	0.55

**Only clinical + MRI features on-treatment**	(Intercept)	-0.75
	Only clinical model	0.83
	Computer extracted MRI features model	0.76
**Only clinical + MRI features + liquid biopsies on-treatment**	(Intercept)	-1.08
	Only clinical model	0.80
	Liquid biopsies model	0.39
	Computer extracted MRI features model	0.68
**Only clinical + liquid biopsies post-treatment**	(Intercept)	-0.62
	Only clinical model	0.82
	Liquid biopsies model	0.67
**Only clinical + MRI features post-treatment**	(Intercept)	-0.59
	Only clinical model	0.82
	Computer extracted MRI features model	0.66
**Only clinical + MRI features + liquid biopsies post-treatment**	(Intercept)	-0.81
	Only clinical model	0.76
	Liquid biopsies model	0.53
	Computer extracted MRI features model	0.36
